# A Couples’ Based Self-Management Program for Heart Failure: Results of a Feasibility Study

**DOI:** 10.3389/fpubh.2016.00171

**Published:** 2016-08-29

**Authors:** Ranak Trivedi, Cindie Slightam, Vincent S. Fan, Ann-Marie Rosland, Karin Nelson, Christine Timko, Steven M. Asch, Steven B. Zeliadt, Paul Heidenreich, Paul L. Hebert, John D. Piette

**Affiliations:** ^1^Stanford University, Stanford, CA, USA; ^2^VA Palo Alto Health Care System, Menlo Park, CA, USA; ^3^VA Puget Sound Health Care System, Seattle, WA, USA; ^4^University of Washington, Seattle, WA, USA; ^5^University of Michigan, Ann Arbor, MI, USA; ^6^VA Ann Arbor Health Care System, Ann Arbor, MI, USA

**Keywords:** dyadic behavior change, couples’ chronic illness, couples’ disease management, caregiver self-management, caregivers

## Abstract

**Background:**

Heart failure (HF) is associated with frequent exacerbations and shortened lifespan. Informal caregivers such as significant others often support self-management in patients with HF. However, existing programs that aim to enhance self-management seldom engage informal caregivers or provide tools that can help alleviate caregiver burden or improve collaboration between patients and their informal caregivers.

**Objective:**

To develop and pilot test a program targeting the needs of self-management support among HF patients as well as their significant others.

**Methods:**

We developed the Dyadic Health Behavior Change model and conducted semi-structured interviews to determine barriers to self-management from various perspectives. Participants’ feedback was used to develop a family-centered self-management program called “SUCCEED: Self-management Using Couples’ Coping EnhancEment in Diseases.” The goals of this program are to improve HF self-management, quality of life, communication within couples, relationship quality, and stress and caregiver burden. We conducted a pilot study with 17 Veterans with HF and their significant others to determine acceptability of the program. We piloted psychosocial surveys at baseline and after participants’ program completion to evaluate change in depressive symptoms, caregiver burden, self-management of HF, communication, quality of relationship, relationship mutuality, and quality of life.

**Results:**

Of the 17 couples, 14 completed at least 1 SUCCEED session. Results showed high acceptability for each of SUCCEED’s sessions. At baseline, patients reported poor quality of life, clinically significant depressive symptoms, and inadequate self-management of HF. After participating in SUCCEED, patients showed improvements in self-management of HF, communication, and relationship quality, while caregivers reported improvements in depressive symptoms and caregiver burden. Quality of life of both patients and significant others declined over time.

**Conclusion:**

In this small pilot study, we showed positive trends with involving significant others in self-management. SUCCEED has the potential of addressing the growing public health problem of HF among patients who receive care from their significant other.

## Introduction

Heart failure (HF) is a significant and growing public health problem characterized by frequent episodes of worsening symptoms, poor quality of life, and shortened lifespan (1–3). Approximately 20% of hospitalized HF patients, >65 years old, are re-hospitalized within 30 days of discharge (4). Between 2010 and 2030, the direct costs related to HF are expected to increase 215%, from $25 billion to nearly $80 billion (5).

To maximize their health and decrease symptom burden, HF patients need to master skills that facilitate self-management, including the ability to: adhere to medication regimens, diet, and physical activity recommendations; communicate with their health-care team; and receive appropriate screening tests and immunizations (6). Multiple studies have demonstrated that following self-management recommendations improve HF patients’ patient-centered health outcomes while reducing hospitalizations, ER visits, and outpatient visits (7–11). HF self-management typically involves ongoing support from members of the health-care team such as nurses. However, many of these interventions do not work. Peikes and colleagues (12) studied 15 nurse-led care coordination programs for Medicare beneficiaries (*N* = 18,309 patients; 48% HF) and found that 13 of the 15 programs did not improve hospitalization rates or measures of adherence. Additional supports may be necessary to assure that patients can attain behavior goals and avoid costly exacerbations.

Patients with greater social resources and especially those who are married have better quality of life, lower depression rates, and longer lifespans (13–16). This is in part due to improved adherence, as family caregivers bridge the gap between health services and self-management activities (17–19). Involving caregivers in disease management improves patients’ quality of life, self-efficacy, and relationship quality (20–22) and reduces hospitalizations in many clinical populations (23–25). The role of spousal caregivers (spouses or significant others who provide care) is especially important. The proximity of spousal caregivers to patients, shared activities of daily living, and established relationships place caregivers in a unique position to influence HF self-management (26).

We developed a couples’ based HF self-management support program called SUCCEED (Self-management Using Couples’ Coping EnhancEment in Diseases). The purpose of this program is to improve communication between HF patients and their significant others and improve self-management. Secondary goals include alleviating stress and improving quality of life for patients and their significant others. In this manuscript, we describe the process that we used to develop the program and report the results of a pilot study designed to determine program acceptability among target participants.

## Materials and Methods

### A Conceptual Model for Dyadic Health Behavior Change

**Figure 1:**
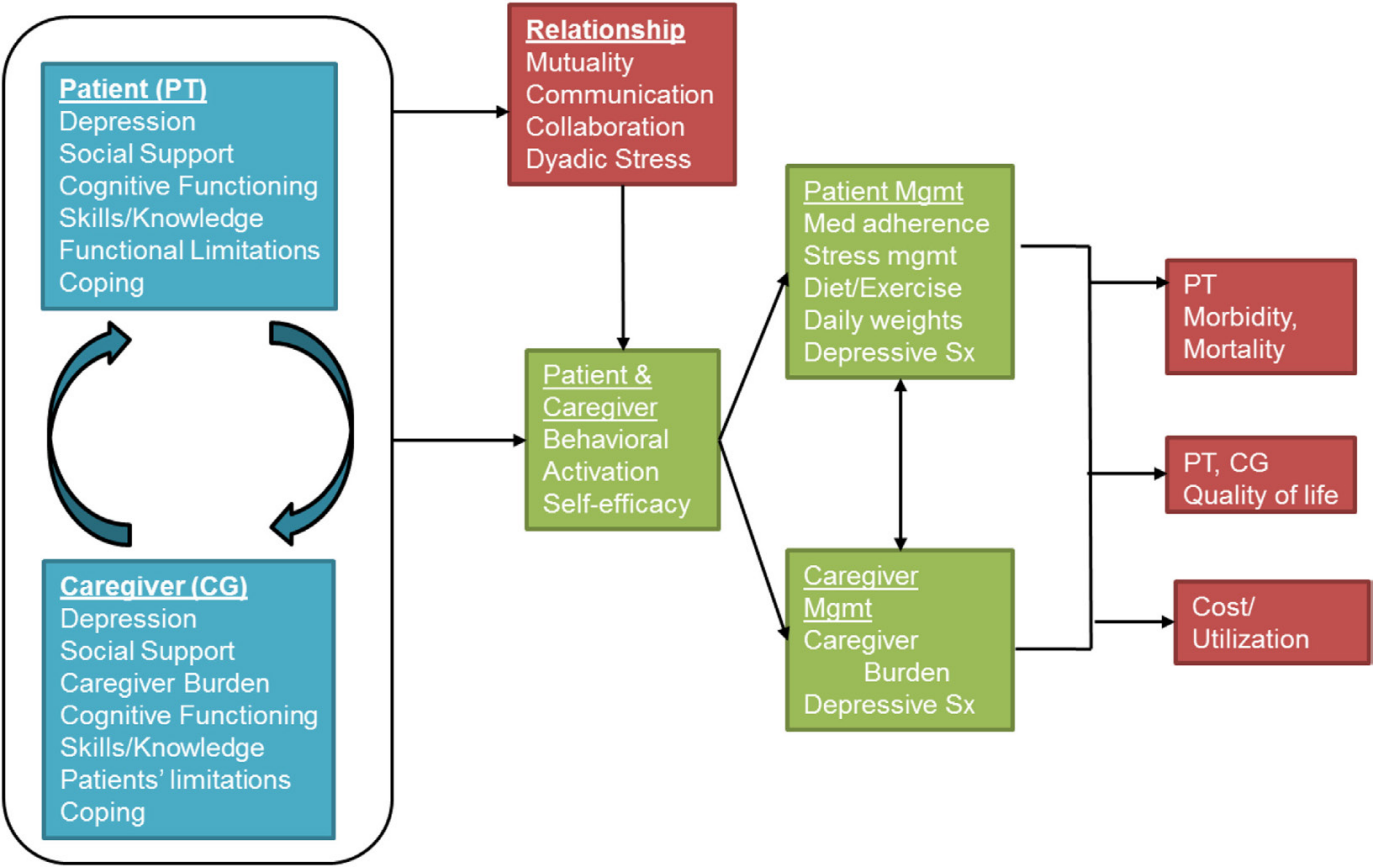
**The Dyadic Health Behavior Change Model**.

Our Dyadic Health Behavior Change model (Figure 1) derives from Leventhal’s Self-regulatory Model of Illness Behavior (27) and Bandura’s Unifying Theory of Self-efficacy (28). These two well-known models demonstrate how improving patients’ self-efficacy can lead to more activated patients, which in turn can improve self-management. Patient activation is defined as having the self-efficacy, motivation, and behavioral skills necessary to self-manage chronic illness, collaborate with health-care providers, maintain function, monitor symptoms, and access appropriate care (29). Patient activation is modifiable (30) and has been linked to a lower probability of having an emergency department visit, being obese or smoking (31, 32). In the conceptual framework motivating the intervention and evaluation plan, we have included corresponding caregiver factors that can influence self-management and clinical outcomes.

A key innovation in our conceptual model is the synergy between the patient and the spousal caregiver, an aspect of disease management neglected in previous theoretical models (33–35). We posit that focusing on reducing individual stress is necessary but insufficient to enhance long-term coping. Interventions should instead build skills around the interactive aspects of coping, including enhanced collaboration and sharing of the responsibilities. We draw on the Dyadic Coping Model (36) to operationalize this synergy and to elucidate pathways linking patient and caregiver individual experiences with relationship outcomes. The Dyadic Coping Model posits that a chronic illness affects both the patients and their spousal caregivers. Because patients and spousal caregivers both contribute to self-management, the quality of their interpersonal relationship may offset individual barriers to self-management. When the relationship is strong and mutual affection exists, spousal caregivers may experience an increase in personal satisfaction when providing care (37).

Relationship quality is a multidimensional construct that may encapsulate happiness, stability, adjustment, communication, coping, and support, and is considered essential for marital harmony (38). Open and reciprocal interactions (“mutuality”) have been shown to facilitate emotional resiliency, positive coping strategies, and better social support (39–41). Conversely, negative interactions marked by criticism, over-involvement or avoidance by caregivers may lead to adverse outcomes in couples (42–44). Many patients report frequent criticism and “nagging” from their family members, leading to lower self-efficacy and poorer self-management (43). Therefore, coping efforts of each partner should focus on the functioning of the other partner as well as the relationship (36). In this way, our model recognizes the individual contributions of both the patient and spousal caregiver while simultaneously recognizing the importance of relationship factors that can influence self-management and ultimately, patient outcomes. Ensuring that patients and spousal caregivers work together may be the optimal strategy to manage chronic illnesses. Self-management interventions that target chronic illnesses should leverage the positive influences that patients and caregivers can have on self-management when the relationship is strong, while ensuring that the negative consequences of poor relationship quality do not impinge on patient health. We published an earlier version of this model (45) where we describe the components and pathways; Figure 1 represents an update of the previous model.

#### Step 1: Initial Development

We conducted semi-structured interviews to identify intervention targets from the perspectives of 17 couples managing HF, and 14 clinical providers at a VA health-care facility. These interviews helped identify key barriers and facilitators of self-management from the perspectives of patients, caregivers, and providers. Barriers included the lack of communication between patients and their caregivers around self-management, and between patients and their providers; lack of information about their disease and how to self-manage; the importance of stress management; and managing interpersonal conflict.

Guided by our theoretical model and results of these interviews, we developed the SUCCEED intervention to specifically address the barriers to self-management, as well as support and maintain those skills which enhance self-management. We accomplished this by adapting and modifying intervention components from three widely used programs:

1. *Stanford Chronic Disease Self-Management Program (CDSMP)* (46): The CDSMP is a cross-disease self-management program that targets multiple behavior. CDSMP addresses three skill sets: skills to manage illness (e.g., managing medications), skills to continue normal life (e.g., maintaining relationships), and skills to cope with negative emotions (e.g., relaxation) (46). CDSMP is co-facilitated by a trained facilitator and a peer, and is delivered in 2.5-h sessions over 6 weeks. CDSMP is widely used and has shown modest effect sizes in short-term and long-term outcomes of quality of life, health, and utilization (11). Like SUCCEED, CDSMP is based on Bandura’s Model of Self-efficacy. *SUCCEED incorporates the CDSMP components of Action Planning, stress management techniques, and building a fulfilling life*.
2. *VA National Caregiver Training Program*: The VA National Caregiver Training Program was developed by the VA Office of Caregiver Support to enhance self-care among caregivers of Veterans. It covers caregiver self-care, home safety, caregiver skills, taking care of Veterans’ personal needs, managing challenging behavior, and resources for self-management. There is considerable overlap between the self-management components laid out by the *patient-focused* CDSMP and the *caregiver-focused* National Caregiver Training Program. *From this program, SUCCEED incorporates the modules relevant to caregiver self-care and managing difficult patients with chronic illness*.
3. *Couples Coping Enhancement Training (CCET) Program* (47): The CCET is based on the Dyadic Coping Model, and facilitates mutual emotional support and dyadic coping among couples where one of the partners has a chronic illness (47, 48). CCET has been tested with over 500 couples from a variety of clinical populations and has been shown to improve marital quality, interpersonal communication, individual coping, and dyadic coping (36, 49). CCET requires in person attendance to a series of six, 3-h group sessions. Shorter versions have been found to be efficacious among breast cancer patients (20, 50). CCET, like CDSMP, is a strong foundation for our program but needed to be adapted from its original format so that it is accessible, feasible, and relevant for patients with HF and their spousal caregivers. *SUCCEED draws on the conceptual framework of CCET. In addition, we have adapted CCET materials relevant to dyadic coping, enhancing collaboration and communication, problem-solving, and enjoying pleasant activities together*.

Standard cognitive behavioral therapy techniques such as problem-solving were incorporated into the intervention. We also obtained written materials for HF self-care support from the former VA CHF Quality Enhancement Research Initiative (QUERI).

#### Step 2: Refining Materials

Our preliminary program and related handouts were reviewed by the local Patient Education Coordinator and Director of Office of Education who suggested modifications to ensure that the materials were at the appropriate reading and education level. We further obtained stakeholder feedback from an existing panel of patients and family members. Their review yielded key modifications, such as limiting the number of sessions to six, and offering couples the option of participating over the telephone.

#### Step 3: Finalizing the Pilot SUCCEED Program

SUCCEED consists of six sessions, with each session addressing new topics and skills for the patient and their caregiver (Table 1). At the end of each session, individuals create an “Action Plan,” in which they note what skills they will practice throughout the week. In each session, couples spend about 5 min reviewing homework and action plans, 35 min learning new material, and 5 min creating a new action plan for the week. Sessions are led by a Masters’ level facilitator who has a health-related graduate degree and has been trained on the SUCCEED program. The session topics are provided next.

**Table 1:** **Description of SUCCEED**.

Sessions	Focus	Source
Session 1: skills to manage disease and caregiver burden	Overview, importance of patient and caregiver self-care	CHF QUERI^a^ (HF specific materials), National Caregiver Training Program (caregiver burden)
Sessions 2 and 3: skills to manage negative emotions	Psychoeducation on the negative impact of illness on both the patient and their spousal caregivers, and how to work as a team to cope with these emotions	CDSMP^b^ (patient-focused strategies), National Caregiver Training Program (caregiver-focused strategies), CCET^c^ (importance of teamwork)
Session 4 and 5: skills to manage interpersonal relationship and relationship stress	Importance of maintaining a strong interpersonal connection and strategies to improve teamwork	CCET
Session 6: building a fulfilling life with HF	Problem-solving pursuit of pleasant activities in the context of barriers endemic to HF; strategies to maintain change	CDSMP

*^a^CHF QUERI: Chronic Heart Failure Quality Enhancement Research Initiative*.

*^b^CDSMP: Chronic Disease Self-Management Program*.

*^c^CCET: Couples Coping Enhancement Training*.

##### Session 1: Skills to Manage HF and Caregiver Burden

The facilitator provides an overview of the program, followed by content specific to HF self-management and caregiver self-care. They define self-management and emphasize its importance for improving HF outcomes. Couples receive education and written materials about managing HF (e.g., reducing salt and fluid intake, daily weights) as well as resources for caregivers in managing HF. The session emphasizes the importance of managing spousal caregivers’ well-being and self-care. The facilitator guides couples in identifying a reasonable and desirable behavior to change and help with goal-setting so that couples have a higher likelihood of success.

##### Sessions 2 and 3: Skills to Manage Negative Emotions

These sessions emphasize the negative emotional impact of HF on both the patient and their spousal caregiver, and how to work as a team to cope with these emotions. Couples receive information on common negative emotions (e.g., depressive symptoms) and how these can increase individual and relationship stress. Information focuses on identifying triggers of negative emotions for patients and their spousal caregiver since these may be different for each person. For example, patients may feel depressed because of their disability and loss of autonomy, whereas spousal caregivers may feel depressed because their loved one is sick or because of increased responsibilities. Couples are taught three stress management strategies: diaphragmatic breathing, progressive muscle relaxation, and guided imagery. Importantly, couples are taught to guide themselves and their partner in these stress management techniques.

##### Sessions 4 and 5: Skills to Manage Interpersonal Relationship Issues and Relationship Stress

These sessions address the importance of maintaining a strong interpersonal connection in the presence of HF or other chronic illness. Strategies address building empathy, increasing constructive communication, reducing negative/counterproductive interactions, improving collaboration, and reframing HF as “our problem” not the “patient’s problem” or “significant other’s problem.” The therapist introduces the concepts of fairness and cost-benefit in the relationship, and s/he works with the couple to problem-solve ways in which both members can contribute to different independent activities of daily living.

##### Session 6: Building a Fulfilling Life with HF

In this session, the facilitator elicits information about hobbies and other pursuits that are enjoyable to patients, spousal caregivers, or both. These may be current activities or activities that the couple used to enjoy before the patient was diagnosed with HF. Exercises focus on identifying these activities and anticipating and addressing barriers to pursuing them. The facilitator focuses on planning the daily routine to accommodate HF needs, anticipating and problem-solving future barriers to self-management, and helpful tips to stay on course. The focus is on practicing existing skills and continuing to work together to set goals as a couple. The protocol re-emphasizes self-management tips from Session 1, since these address short-term and long-term barriers. Information related to community resources and VA programs (e.g., respite care) is provided.

#### Step 4: Pilot Test of SUCCEED

The pilot study was designed to pilot recruitment and retention procedures, evaluate psychosocial measures, and obtain measures of acceptability and feasibility. This study was approved by and carried out in accordance with the recommendations of the IRB at VA Palo Alto Health Care System and Stanford University with written informed consent from all participants.

##### Methods

- *Screening and Enrollment*: Patients were identified *via* registries from the VA Decision Support System (DSS) and VA Palo Alto Heart Failure Clinic research registry. These registries included a list of patients with a diagnosis of HF (ICD-9 code 428.XX), and who had 1+ visit to the local facility in the past year. Participants were eligible if they had been seen for HF within the past year; were not actively receiving chemotherapy for cancer, were not on hemodialysis; did not have dementia or other cognitive impairments; had a primary caregiver who was a spouse or significant other; and did not have paid caregiver support. Detailed records about the recruitment process were maintained including the total number of couples screened, number of couples contacted, number of eligible versus non-eligible couples, reasons for ineligibility, and reasons for non-participation among eligible couples. Once enrolled, the research assistant would maintain regular contact with participants *via* phone and send material *via* trackable mail to ensure that they were received. Dropout was minimized by rescheduling missed appointments. Patients who were hospitalized remained enrolled unless they requested a withdrawal.
- *Intervention Delivery*: The SUCCEED program was provided by phone to Veterans and their spousal caregivers through the six sessions described above. Each session was 45–60 min in duration. Dr. Trivedi, the first author, trained the facilitators to deliver the program through in person sessions and practicing. All written materials were mailed to the couples ahead of time and duplicates were sent as necessary. Dr. Trivedi periodically reviewed the audio recordings of sessions and provided corrective feedback as needed to ensure the integrity of the program.
- *Psychosocial Surveys*: We obtained demographic information at baseline, and piloted psychosocial surveys at baseline and follow-up that measured constructs identified in our conceptual model. For participants who did not complete all six sessions, we requested receipt of the follow-up surveys. Both members of the couple completed the Medical Outcomes Study Short Form-12 version 2.0 (SF-12), which measures quality of life (51), the Patient Health Questionnaire (PHQ-9) (52), which measures depressive symptoms (scores ≥10 indicate clinically significant depressive symptoms), the Dyadic Coping Inventory (53), which measures collective coping and relationship quality (scores ≥100 indicate a strong relationship), the Mutuality Psychological Development Questionnaire (54), which measures reciprocity in the relationship, and the Couples’ Illness Communication Scale (55), which measures communication within the couple around a chronic condition. The SF-12 provides a Physical Component Summary (PCS) score and a Mental Component Summary (MCS) score, with lower scores indicating worse quality of life. Both scales are standardized with a mean of 50 and SD of 10. Patients additionally completed the Minnesota Living with Heart Failure Questionnaire (56), which measures HF specific quality of life and the Self-care of Heart Failure Index v6 (57) which measures patients’ ability to self-manage HF. The Self-care of Heart Failure Index consists of 22 items and 3 subscales: self-care maintenance (10 items), self-care management (5 items), and self-care confidence (6 items). Scores ≥70 on each scale indicate adequate HF self-management; changes in scores predict ED visits, hospitalizations, and mortality (57). Significant others additionally completed the Caregiver Reaction Assessment (58), which measures the positive and negative aspects of caregiving.
- *Participant Feedback*: We assessed acceptability after each session of SUCCEED *via* mailed feedback surveys asking each participant to separately rate: the extent to which they felt that the objectives of the session were reasonable, the objectives were met, the homework assigned was relevant, they felt that they learned something, and they believed they would use what they had learned. Each item was scored on a five-point Likert scale on which higher scores indicated greater acceptability. We aggregated the ratings across all participants (patients and caregivers). We also conducted an exit interview at the end of the program or when participants withdrew to obtain feedback on the program, assess barriers to completing surveys, and reasons for withdrawal if appropriate.
- *Facilitator Feedback*: After each session, facilitators recorded their experience delivering the content or any suggestions or concerns expressed by couples during sessions.

##### Data Analyses

Descriptive analyses were conducted at baseline and at the end of treatment. Unadjusted change scores were generated for all participants with pre and post-data. Unadjusted Pearson’s product moment correlations were conducted with continuous scores on the psychosocial surveys.

##### Results of Pilot Study

**Figure 2:**
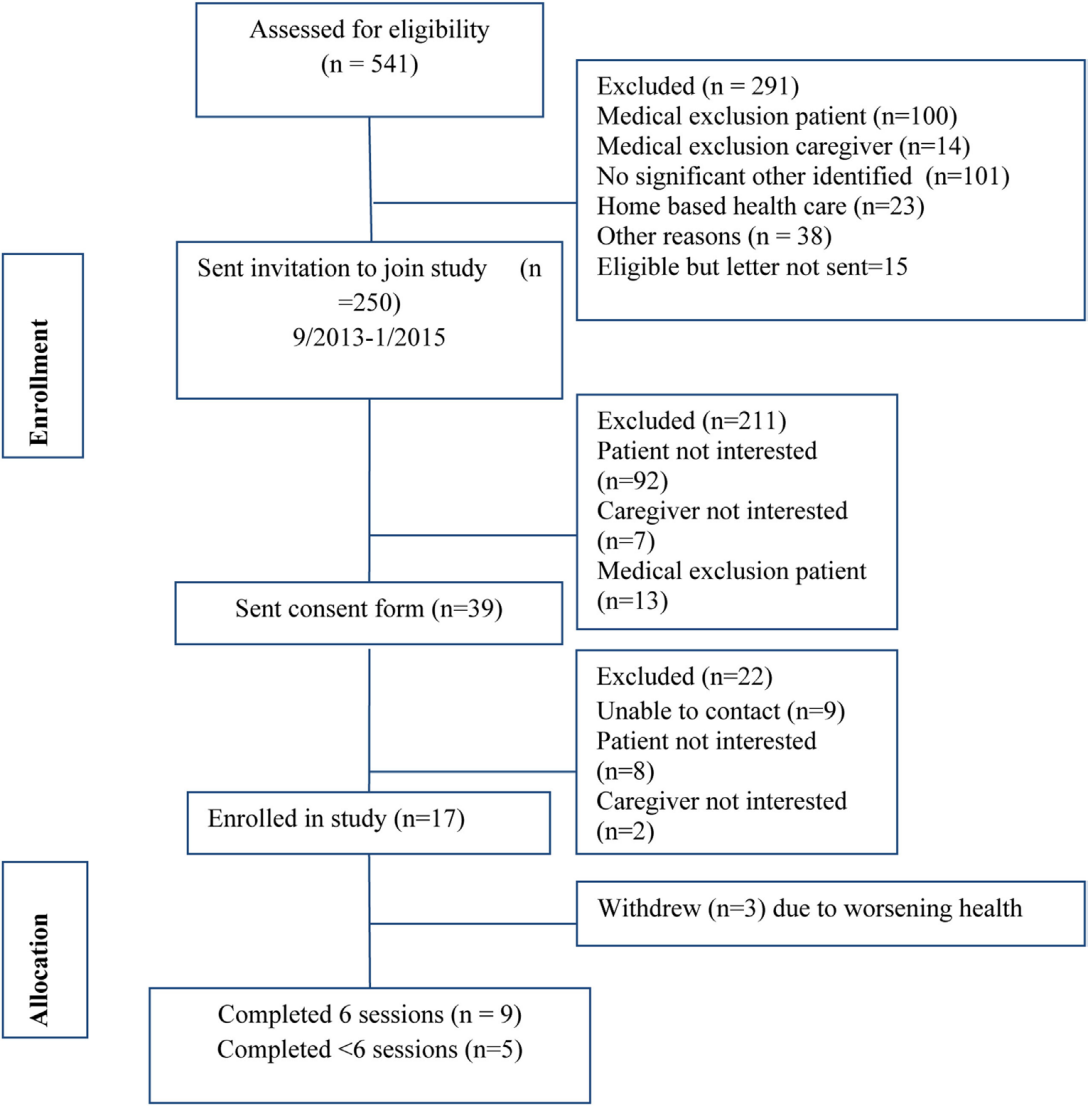
**Recruitment diagram**.

The recruitment rate was 6.8% of all eligible patients (Figure 2).

Seventeen couples were enrolled in the pilot. Nine couples completed all six sessions while five completed at least one session; three withdrew prior to the first session citing deteriorating health of the patients. Table 2 describes the demographics of participants who completed the first study session. The mean age of patients was 68.4 (SD = 11.3) and of significant others was 64.4 (SD = 11.0). The sample was 78.6% White, 71% had some college education, and 21% of patients and 28.6% of spouses were working either part or full time. Participants felt financially constrained, as only 28.6% reported that they were “able to pay the bills and have money left over for special things.”

**Table 2:** **Baseline characteristics of participants who completed psychosocial surveys**.

	Patient (*n* = 14)	Caregiver (*n* = 14)
**Age**	68.4 (11.3)	64.45 (11)
**Ethnicity (*n*)**		
White	11	11
Black	1	1
Native American	1	0
More than one race	1	2
**Hispanic (*n*)**	0	4
**Education (*n*)**		
HS	3	4
Some College or Degree	10	10
Graduate school	1	0
**Employment (*n*)**		
Full-time	2	3
Part-time	1	1
Retired	8	5
Not employed	3	4
Homemaker	0	1
**Years since diagnosis, M (SD)**	5.1 (4.7)	
**#Conditions, M (SD)**	8.1 (2.3)	2.7 (2.4)
**Finances (*n*)**		
Can pay bills with extra for special things	4	4
Can pay bills, but little spare money for extra	6	7
Can pay the bills but must cut back	3	3
Have difficulty paying the bills	1	

##### Feasibility and Acceptability

Our key measures of feasibility were recruitment and retention. A detailed recruitment diagram is available in Figure 2. During the study, recruitment methods were refined to enhance enrollment and manage the challenges of a mail and phone based study. For instance, the first 180 invitation letters required the patients to contact us if they wanted to learn more about the study. With this method, we were not allowed to have further contact with patients unless they contacted us. This method resulted in recruitment challenges such that in 8 months only eight patients were recruited. We changed our recruitment methodology such that patients could contact us if they did not want to participate. If we did not receive such information, the research assistant would follow up by phone to assess if letters were received and if the patient and/or spousal caregiver were interested in learning more about the study. By modifying this method, we were able to improve our recruitment rate and improve efficiency.

**Figure 3:**
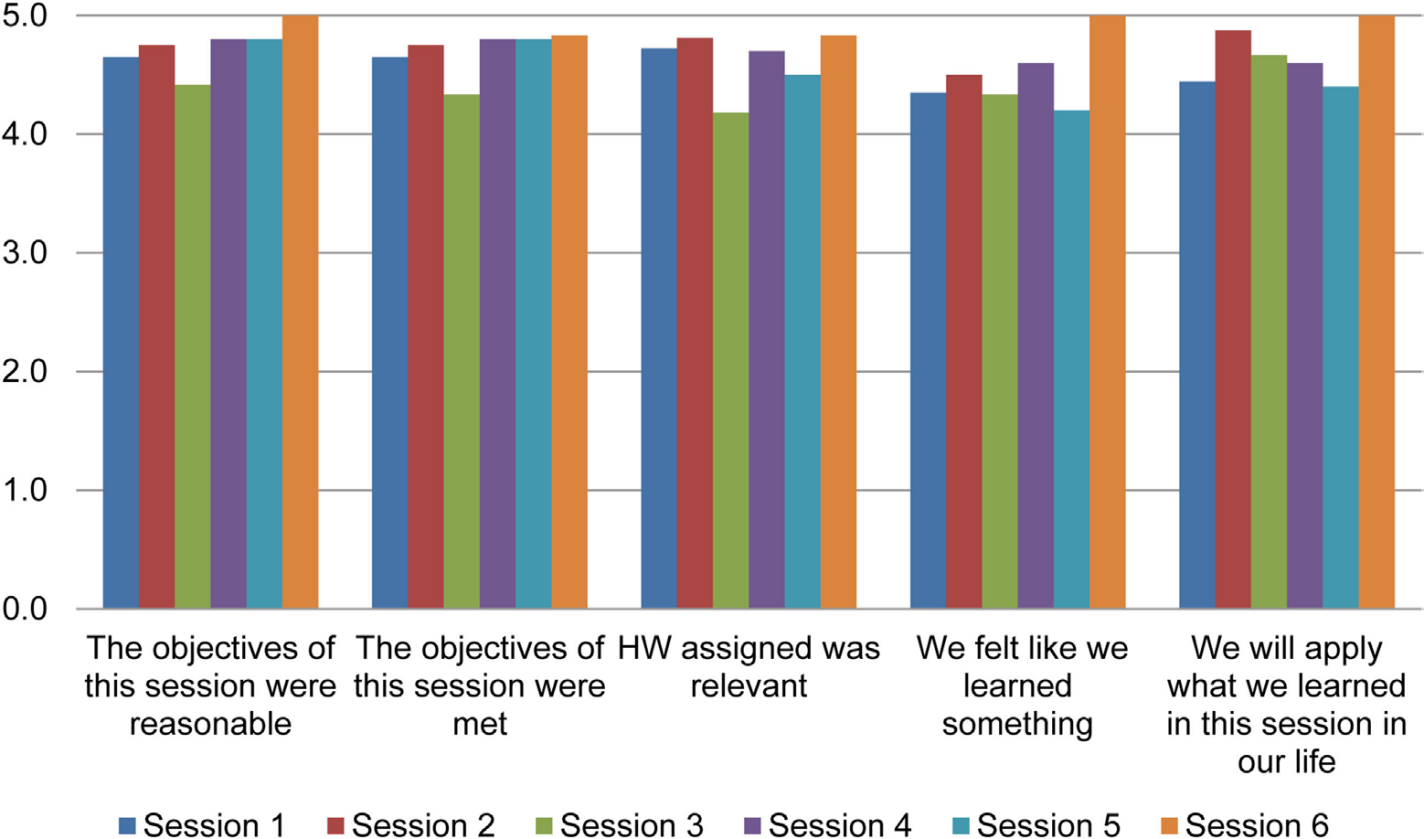
**Acceptability data**.

The additional nine couples were enrolled within 6 months, and 19 more couples were interested but could not be recruited due to budgetary constraints on the part of the project. As can be seen in Figure 3, session acceptability ratings ranged a mean of 4.2 (low) to 5 (high), indicating patients and significant others both reported high acceptability of the sessions.

The average time for completing all six SUCCEED sessions was 11 weeks. The average time between pretest and posttest measurements was 13.7 weeks for completers of SUCCEED and 12.9 weeks for non-completers. Participants who did not complete the study were slightly older than completers, and fewer of them were married. There were fewer White caregivers among non-completers but a higher proportion of completers were Hispanic. No other consistent trends were seen. Table 3 shows the baseline and follow-up scores on the psychosocial measures. On average, patients were depressed at baseline (PHQ9 = 11.1 ± 6.8) whereas significant others were not (PHQ9 = 5.6 ± 4.2). At baseline, patients reported adequate self-care maintenance (71.7 ± 9.9) but less-than-adequate self-care management (57.9 ± 19.3) and self-care confidence (63.3 ± 17.6). At baseline, both patients (140.4 ± 18.2) and significant others (141.9 ± 23.8) reported high scores on the Dyadic Coping Inventory. Patients were approximately 1 SD below the average on physical quality of life (PCS = 39.3 ± 7.3) and approximately 0.5 SD below the average on mental quality of life (MCS = 45.6 ± 7.8). Significant others reported 1 SD lower mental quality of life (MCS = 39.8 ± 8.0) and.5 SD lower physical quality of life (PCS = 46.2 ± 11.0).

**Table 3:** **Unadjusted change scores for pilot study participants, M (SD)**.

Measure	Desired change	Patient		Caregiver	
		Pre	Post	Pre	Post
Patient Health Questionnaire-9	Lower	11.1 (6.8)	11.0 (7.1)	5.6 (4.2)	4.7 (4.9)
Caregiver reaction assessment	Higher	–	–	65.1 (8.9)	67.0 (11.4)
Self-care of heart failure index	Higher				
*Maintenance*		71.7 (9.9)	74.2 (9.4)	–	–
*Management*		57.9 (19.3)	61.4 (19.7)	–	–
*Confidence*		63.3 (17.6)	69.5 (10.7)	–	–
Dyadic coping inventory	Higher	140.4 (18.2)	142.9 (16.5)	141.9 (23.8)	136.3 (13.9)
Couples’ illness communication scale	Higher	14.5 (3.5)	15.8 (3.2)	15.6 (3.5)	14.7 (3.9)
Mutuality psychological development questionnaire	Higher	76.2 (5.1)	77.2 (2.4)	69.3 (5.9)	73.6 (2.2)
Minnesota living with HF questionnaire	Lower	50.9 (31.9)	56.1 (28.1)	–	–
SF-12	Higher				
*Physical component*		39.3 (7.3)	33.5 (4.7)	46.2 (11.0)	39.4 (8.9)
*Mental component*		45.6 (7.8)	34.3 (10.3)	39.8 (8.0)	33.3 (7.93)

Following participation in SUCCEED, caregivers showed desired changes for depressive symptoms (PHQ9, 5.6 vs. 4.7), caregiver burden (Caregiver Reaction Assessment, 65.1 vs. 67), and mutuality (Mutuality Psychological Development Questionnaire, 69.3 vs. 73.6). Patients showed improvements in all three subscales of the Self-care of HF Index: self-management maintenance (71.7 vs. 74.9), management (57.9 vs. 61.4), and confidence (63.3 vs. 69.5). In addition, patients also showed slight improvements in relationship quality (Dyadic Coping Inventory, 140.4 vs. 142.9), mutuality (Mutuality Psychological Development Questionnaire, 76.2 vs. 77.2), and communication (Couples’ Illness Communication Scale, 14.5 vs. 15.8). Patients’ depressive symptoms did not change (11.1 vs. 11.0).

These positive changes were despite the decline in quality of life for patients and their caregivers. As can be seen, patients’ quality of life worsened across the disease specific measure (Minnesota Living with Heart Failure Questionnaire, 50.9 vs. 56.1), the general measure of physical well-being [SF-12 PCS score, 39.3 vs. 33.5], and the general measure of mental well-being [SF-12 MCS score, 45.6 vs. 34.3]. Similarly, the caregivers’ quality of life also declined, as evidence by worsening physical well-being (46.2 vs. 39.4) and mental well-being (39.8 vs. 33.3). Caregivers noted worsening communication (Couples’ Illness Communication Scale, 15.6 vs. 14.7). Caregivers also noted a decrement in dyadic coping (Dyadic Coping Inventory, 141.9 vs. 136.3).

Results of simple correlations suggested a few notable associations. The full table is provided in Supplementary Material. Better communication as reported by patients at baseline and follow-up was associated with better HF specific quality of life (*r* = −0.73 at baseline, *r* = −0.72 at follow-up, both *p*’s <0.05). Better relationship quality as reported by patients was associated with patients’ HF self-care maintenance (*r* = 0.69, *p* < 0.05) and management (*r* = 0.74, *p* < 0.05). At baseline, caregivers’ report of mutuality was negatively correlated with their own depression (*r* = −0.82, *p* < 0.01), as well as patient’s depression at baseline (*r* = −0.85, *p* < 0.01) and follow-up (*r* = −0.91, *p* < 0.01). Interestingly, patients’ and caregivers’ report of relationship quality were not significantly correlated at either baseline or follow-up.

## Discussion

This study found that a theoretically derived family-centered self-management program is feasible and highly favored by patients and their significant others. The self-management program engaged both the patient and the significant other in an effort to increase teamwork around self-management recommendations. At baseline, the quality of relationships was very strong in our sample. Patients reported mild depressive symptoms on average, and caregivers were not depressed. The general quality of life, including physical and mental well-being, was below average at baseline for both patients and their caregivers.

The largest positive effects of SUCCEED were seen in patients’ report of self-management of HF, as indicated by the subscales of the Self-care of HF Index. As noted earlier, scores above 70 are indicative of adequate self-management, and improvements of five points or more are associated with improved clinical outcomes (57). At baseline, scores on the Self-care Management and Self-care Confidence scales indicated inadequate self-management. Following SUCCEED, the scores on the Self-care Confidence increased 6.2 points, and those for Self-care Confidence increased 3.5 points. Even the scores on Self-care Maintenance, which indicated adequate self-care at baseline, improved 2.5 points. Therefore, in this small pilot grant, SUCCEED appeared to be successful at achieving one of its key goals which was to improve self-management of HF.

Another important goal of SUCCEED was to improve the relationship between patients and their spousal caregivers. Both patients and caregivers reported improvements in mutuality, which is a measure of empathy in the relationship. This is important because we devoted much of Session four to empathy building through exercises such as active listening. In contrast, patients and caregivers appeared to have opposite results on coping and communication. While patients showed slight improvements, caregivers showed slight decrements. It was noted that caregivers’ post-intervention scores on dyadic coping still indicated a strong relationship. One possible explanation is that for caregivers, their scores on relationship quality had a ceiling effect. Collectively, these results suggest that SUCCEED may improve empathy for both members of the patient-caregiver dyad, but only improve communication and coping for patients.

By contrast, quality of life was worse for both patients and caregivers following SUCCEED participation. Patients reported worsening disease specific quality of life, as well as worsening general quality of life. Similarly, caregivers also experienced worsening general quality of life. It is possible that participating in SUCCEED added burden for our participants, who were already experiencing poor quality of life at baseline. We are in the process of adapting SUCCEED to be delivered over the web in a self-study format, to allow interested parties to access the material at their own convenience. It is also possible that the worse quality of life was reflective of worsening health over time in this sick population. During the course of our study, one patient died, one was hospitalized, and one withdrew due to a new cancer diagnosis, and two withdrew due to other exacerbating health problems. Family-centered self-management programs should account for the disease severity of the clinical population, and ensure that the programs are not burdensome to participants.

An important goal of our pilot study was to finalize optimal recruitment and retention strategies for dyads managing HF. Recruitment of HF patients into self-management programs is challenging, as evidenced by a recent trial that showed a 5% recruitment rate (59). The recruitment of dyads is even more difficult because it requires significantly more resources and time than recruiting patients alone (60). A recently completed trial by Piette et al. (61) of patient–caregiver dyads also showed a recruitment rate of approximately 5%. Our low retention rate was partly due to the challenges of the clinical population. In one instance, the patient was diagnosed with prostate cancer during the course of the study and requested withdrawal from study participation. Couples who withdrew and underwent exit interviews continued to express support for the program despite their own inability to continue their participation. During the course of our study, we modified our invitation process, improved the invitation materials, and increased the amount of personnel. Our eventual recruitment rate of 5.4% exceeded the recruitment rates of the large-scale trials cited earlier, thus demonstrating the feasibility of our program. Adding a facilitator allowed the project coordinator to devote more time to recruitment and retention, resulting in improvements in both. In a future trial, we will use the methods developed in this pilot study to ensure its success.

SUCCEED is anchored within the successes of other family-centered self-management interventions. A series of studies conducted by Piette and colleagues has shown that engaging a non-cohabitating caregiver in an automated telemonitoring program can enhance adherence to self-management recommendations among patients with heart failure and diabetes (62–64). Across three trials, patients who participated in an interactive voice response based support program benefited more if they engaged a caregiver (61, 65–67). Among patients with HF, patients for whom their caregiver received additional support had better medication adherence, less shortness of breath, and if depressed at baseline, lower depression symptoms compared to those patients for whom their caregiver did not receive such assistance.

A recent AARP report shows that 50 million Americans are informal caregivers and provide $470 billion worth of unpaid services each year (36, 37). Of these, 40.4 million are caregivers of adults ages 65 and older (38). The same report showed that only 16% caregivers have been asked about their own self-care, and more than 80% of caregivers felt they needed tools to manage their own stress. Without the types of assistance that SUCCEED can provide, some of those caregivers may provide less support or leave the caregiving relationship to the detriment of their patient’s longer term health (10, 39). Yet, programs that aim to enhance self-management almost exclusively focus on the needs of patients. Supporting caregivers in their task of caring for the patients and capitalizing on their existing relationships will require systematic policy shifts and investments on the part of the health systems. Otherwise, caregivers are likely to burn out, and are at a higher risk of poor quality of life, depression, and mortality (68). To be sure, many caregivers experience positive emotions as they provide care to their loved ones in need (69). Developing programs and policies that support both of these scenarios – providing tools to enhance positive feelings while decreasing the negative emotions – should remain a strong priority for health systems and policy makers.

The primary limitation of our study was its small sample size, which precluded reliable interpretation of the change scores. A larger sample would be necessary to understand the influence of SUCCEED on key outcomes, as well as to understand mediators and moderators of these relationships. Another limitation is that current results may not be generalizable to non-Veteran patients or non-heterosexual couples. Finally, it is possible that couples who participated in this study were different from those who did not, for example, because they had a stronger and more loving relationship than those who did not participate. Our planned larger studies will address these limitations.

Nevertheless, our results suggest that SUCCEED can simultaneously improve the individual and interpersonal functioning of patients and their caregivers. An adequately powered study will allow us to expand on these positive trends, while allowing us to explore the seemingly detrimental aspects of SUCCEED. Because our main goal was to develop, refine and test this program, we believe that the small sample was sufficient to help us develop the methodology for a larger study. We are encouraged that our program was acceptable to patients and their significant others, in large part due to the ongoing engagement of key stakeholders. This pilot study provides impetus to developing family-centered self-management programs for HF.

## Author Contributions

RT was involved in conceptualization, intervention development, pilot testing, analyses, and manuscript draft. CS was involved in intervention development, analyses, and manuscript draft. JP, VF, A-MR, and KN were involved in conceptualization, intervention development, and manuscript draft. SZ, PLH, PH, CT, and SA were involved in pilot testing, and manuscript draft.

## Conflict of Interest Statement

The authors declare that the research was conducted in the absence of any commercial or financial relationships that could be construed as a potential conflict of interest.
