# Supplementary material for: A Couples’ Based Self-Management Program for Heart Failure: Results of a Feasibility Study
Source: Front Public Health. 2016 Aug 29;4:171. doi: 10.3389/fpubh.2016.00171 (PMC5004799; doi:10.3389/fpubh.2016.00171)
Supplement: Supplementary file 1 [file Table_1.DOCX]

**Table S1: Demographic Characteristics of Completers (n=9) and Non-completers (n=6).**

|  | **Patient** | | **Caregivers** | |
| --- | --- | --- | --- | --- |
|  | **Completers** | **Non-completers** | **Completers** | **Non-completers** |
| **Age** | 67.1(12.3) | 70.69(10.2) | 62.8(11.3) | 67.3(10.8) |
| **Married %** | 88.9 | 80 | 88.9 | 80 |
| **Ethnicity %** |  |  |  |  |
| White | 77.8 | 80 | 88.9 | 60 |
| African American | - | 20 | - | 20 |
| Native American | 11.1 | - | - | - |
| More than one race | 11.1 | - | 11.1 | 20 |
| **Hispanic %** | 22 | 0 | 66.7 | 20 |
| **Education %** |  |  |  |  |
| Some HS | - | 20 | 11.1 | 20 |
| HS Diploma | 11.1 | 20 | 11.1 | 20 |
| Some College | 88.9 | 40 | 77.8 | 40 |
| Graduate School |  | 20 |  | 20 |
| **Employment %** |  |  |  |  |
| Full-Time | 11.1 | 20 | 22.2 | 20 |
| Part-time | 11.1 | - | - | 20 |
| Retired | 66.7 | 40 | 44.4 | 20 |
| Not employed | 11.1 | 40 | 22.2 | 40 |
| Homemaker |  |  | 11.1 |  |
| **Finances** |  |  |  |  |
| Can pay bill with extra for special things | 22.2 | 40 | 22.2 | 40 |
| Can pay bills but little spare money for extra | 66.7 | 0 | 44.4 | 60 |
| Can pay the bills but must cut back | 11.1 | 40 | 33.3 | 0 |
| Have difficulty paying the bills | 0 | 20 | 0 | 0 |
